# Angiogenesis, Lymphangiogenesis, and the Immune Response in South African Preeclamptic Women Receiving HAART

**DOI:** 10.3390/ijms20153728

**Published:** 2019-07-30

**Authors:** Thajasvarie Naicker, Wendy N. Phoswa, Onankoy A. Onyangunga, Premjith Gathiram, Jagidesa Moodley

**Affiliations:** 1 Optics and Imaging Centre, Doris Duke Medical Research Institute, University of KwaZulu-Natal, Durban 4013, South Africa; 2 Discipline of Obstetrics and Gynecology, Nelson R Mandela School of Medicine, University of KwaZulu-Natal, Durban 4013, South Africa; 3 Women’s Health and HIV Research Group. Department of Obstetrics and Gynecology, School of Clinical Medicine, University of KwaZulu-Natal, Durban 4013, South Africa

**Keywords:** angiogenesis, highly active anti-retroviral therapy, human immunodeficiency virus, lymphangiogenesis, immune response, preeclampsia

## Abstract

**Purpose of the review:** This review highlights the role of angiogenesis, lymphangiogenesis, and immune markers in human immunodeficiency virus (HIV)-associated preeclamptic (PE) pregnancies in an attempt to unravel the mysteries underlying the duality of both conditions in South Africa. **Recent findings:** Studies demonstrate that HIV-infected pregnant women develop PE at a lower frequency than uninfected women. In contrast, women receiving highly active anti-retroviral therapy (HAART) are more inclined to develop PE, stemming from an imbalance of angiogenesis, lymphangiogenesis, and immune response. **Summary:** In view of the paradoxical effect of HIV infection on PE development, this study examines angiogenesis, lymphangiogenesis, and immune markers in the highly HIV endemic area of KwaZulu-Natal. We believe that HAART re-constitutes the immune response in PE, thereby predisposing women to PE development. This susceptibility is due to an imbalance in the angiogenic/lymphangiogenic/immune response as compared to normotensive pregnant women. Further large-scale studies are urgently required to investigate the effect of the duration of HAART on PE development.

## 1. Problem Identification

### Maternal Mortality and Hypertension in South Africa

The adoption of the Millennium Development Goals from 1990–2015 led to a decline in global maternal mortality by 44%; however, South Africa (SA) was unable to reach the target set by the United Nations (Millenium Development Goals, 2015 Report). South Africa has since embraced the Sustainable Development Goals 2016–2030 to reduce its maternal mortality ratio to <70 deaths/100,000 live births [1]. Despite a decline in maternal deaths from human immunodeficiency virus (HIV) infection and obstetric hemorrhage over the period 2008–2016, no change in mortality emanating from hypertensive diseases in pregnancy (HDP) occurred [2]. In fact, deaths from HDP is the commonest direct cause of maternal mortality as reported by the Confidential Report of Saving Mothers in 2017 [2]. Hypertensive diseases in pregnancy account for 18% of all maternal deaths in SA [3]. In developed countries, HDP has a prevalence of 5–10% [4]; however, in developing countries, it occurs more frequently. The incidence of preeclampsia (PE) was 12% amongst all primigravidae who delivered at a large regional hospital in SA [5]. In SA, PE significantly affects both the mother and perinatal morbidity and death. The World Health Organization (WHO) reported that this multisystem pregnancy disorder accounts for 1.6% of maternal deaths in developed countries [6] and 1.8–16.7% in developing countries such as South Africa, Egypt, Tanzania, and Ethiopia [7,8].

## 2. Human Immunodeficiency Virus Infection in South Africa

HIV infection is a grave public health challenge globally. Sub-Saharan Africa constitutes 56% of the HIV-infected global population [9]. In 2017, women accounted for a disparate 59% of new adult HIV infections (>15 years) [10]. In SA, 13.1% of the total population is HIV-positive, of which 20% involves women in their childbearing age (15–49 years) [11]. Greater than 40% of the global HIV-infected population includes adults residing in the region of KwaZulu-Natal (KZN) [9]. Moreover, the Antenatal HIV and Syphilis Surveillance Report indicates that >37% of antenatal attendees in KZN province are infected [12]. Hence, healthcare professionals providing maternity care are challenged with a double burden of HIV infection and HDP.

The association between HIV infection and PE emanates from the different immune responses [13]. In light of the pervasive nature of both conditions in KZN, this association warrants urgent investigation. Notably, in SA, our group performed extensive research on the effect of angiogenesis and lymphangiogenesis in HIV-infected PE women. Therefore, this review serves to highlight the effect of pregnancy type and HIV status on angiogenesis and lymphangiogenesis using South African cohorts. We also provide compelling evidence of the mechanism(s) that HIV utilizes to exploit the angiogenic system. Furthermore, we provide data based on highly active anti-retroviral treatment (HAART) on reconstituting the immune system and its influence on PE development.

## 3. Angiogenesis

Angiogenesis is defined as the migration, development, and differentiation of endothelial cells to form new blood vessels [14]. It is initiated by pro-angiogenic vascular endothelial growth factors (VEGFs) and placental growth factors (PlGFs), which increase vessel permeability and promote proteolysis of the extracellular matrix via proteases, resulting in endothelial cell proliferation. Thereafter, endothelial cells migrate and invade the lumen, followed by endothelial maturation [15,16].

In normal pregnancy, the need for increased blood supply to the fetus is met by the physiological transformation of spiral arteries in both the decidua and myometrium. In contrast, as a result of deficient trophoblast invasion, spiral artery remodeling is restricted to the decidua in PE [17] and is often associated with adverse birth outcome.

Angiogenesis is also dysregulated in HIV-1 infected patients [18]. Notably, adverse birth outcome is elevated upon receipt of anti-retroviral therapy (ART) compared to HIV-uninfected women [19,20]. Since SA has the largest anti-retroviral rollout in the world, it is important to recognize any link(s) between HAART usage in pregnancy and the risk for PE development. In a novel study, Powis et al. (2013) assessed angiogenesis in preeclamptic women that initiated HAART during pregnancy [21]. They demonstrated that women who developed PE had an upregulation of anti-angiogenic factors prior to HAART usage. Moreover, a recent report correlated altered angiogenesis with ARV usage in the second and third trimesters as a progenitor of preterm birth, small for gestational age, and stillbirth [22].

### 3.1. Soluble Fms-Like Tyrosine Kinase 1 (sFlt1), Placental Growth Factor (PlGF), and Soluble Endoglin (Eng) 

It is well documented that placental sFlt1 is elevated in PE, resulting in a rise in systemic levels with a concomitant decline in VEGF and PlGF [23]. The anti-angiogenic factor sFlt-1 is a scavenger receptor for VEGF and PlGF, thereby dampening their constructive effects on the maternal endothelium [24]. Moreover, in pregnant rats, the administration of sFlt1 induces the clinical symptoms of PE [25]. Flt-1 and sFlt-1 levels in the placenta are upregulated in PE compared to controls, irrespective of HIV infection [26]. Working in our laboratory, Govender et al. (2013) demonstrated increasing levels of serum sFlt1 and sEng in PE, regardless of HIV infection [27]. sFlt1 and sEng are implicated in the endothelial dysfunction of PE. Moreover the downregulation of serum sFlt1 and sEng within HIV-infected women advocates counterbalance of the immune hyperactivity in PE [27]. sEng weakens the binding of TGF-β1 to its receptors and blocks the activation of the endothelial nitric oxide synthase 3 (eNOS) pathways downstream, thereby inducing hypertension [28]. The recent use of sFlt-1:PlGF ratio for the clinical prediction of severe early-onset PE is encouraging [29].

### 3.2. Vascular Endothelial Growth Factor (VEGF)

The permeability of blood vessels is enhanced by VEGF, thereby inducing angiogenesis and vasculogenesis [30]. The VEGF family comprises VEGF-A, VEGF-B, VEGF-C, VEGF-D, and PlGF [31]. VEGF receptors include VEGFR-1 (Flt-1) and VEGFR-2 (Flk-1/KDR) [31]. VEGF-A and VEGF-B bind to VEGFR-1 (Flt-1); however, in PE, binding is blocked by the antagonist sFlt-1 or sVEGFR-1, a spliced soluble variant of VEGFR-1 [32]. VEGFR-2 is an antagonist to VEGF and increases arterial pressure [33]. Both VEGF-C and VEGF-D bind to VEGFR-3, thus expediting lymphangiogenesis [34].

### 3.3. Platelet Endothelial Cell Adhesion Molecule *1 (PECAM-1)*

Vascular development is influenced by PECAM-1 through the formation of a complex with VEGFR-2 and VE cadherin [35]. In PE, PECAM-1 induces neutrophil and platelet activation, thereby promoting vascular damage [36]. Thakoordeen et al. (2017) demonstrated a similar level of PECAM-1 between control and preeclamptic pregnancies (*p* = 0.07), while no correlation was found based on HIV infection (*p* = 0.68) or across study groups (*p* = 0.24) [37].

### 3.4. Angiopoietin (Ang)-2

The angiopoietin family includes Ang-1, Ang-2, Ang-3, and Ang-4 types, which are vital for embryonic angiogenesis. These growth factors are ligands for the vascular endothelial receptor tyrosine kinase (Tie-2), required for vascular activation [38]. Mbhele et al. (2017) demonstrated that, in contrast to PlGF, increased levels of Ang-2 and Eng were noted in PE. The gestational period (early- or late-onset PE) had no effect on Ang-2 expression; yet, it was associated with Eng (*p* < 0.0001) and PlGF (*p* = 0.0033). HIV infection did not affect Ang-2 (*p* = 0.4), Eng (*p* = 0.4), and PlGF (*p* = 0.7) levels [39].

### 3.5. sTie-2

During development, vascular endothelial cells express the transmembrane tyrosine kinase receptors Tie-1 and Tie-2, which are responsible for vascular maturation and angiogenesis [40]. Angiopoietin-1 via Tie-2 signaling facilitates endothelial development, whilst Ang-2 acts as an Ang-1 antagonist by binding to the Tie-2 receptor [41].

However, whilst vessel growth is dependent on Tie-2 [19], Tie-2 may be proteolytically cleaved to produce sTie-2. This soluble form inhibits Tie-2 signaling by averting angiogenesis [19,20]. Mazibuko et al. (2019) demonstrated that soluble Tie-2 levels were dissimilar between preeclamptic and control pregnancies (*p =* 0.0403). In contrast, HIV status did not affect sTie2 and soluble human epidermal growth factor receptor 2 (sHER2) manifestation [42]. Also, HER2 is a membrane-bound receptor tyrosine kinase that is shed via proteolytic cleavage into body fluids [43]. Mazibuko et al. (2019) reported that sHER2 levels were similar between pregnancy types (control *vs.* PE; *p =* 0.3677), regardless of HIV status (*p =* 0.5249). These results may be due to the hypoxic pro-oxidative milieu of both PE and HIV infection, as sHER2 interferes with mitogen-activated protein kinase (MAPK) and Phosphatidylinositol-3-kinase/ protein kinase B (P13K/Akt) signaling [42].

### 3.6. Vascular Endothelial Growth Factor and HIV Tat protein

The accessory protein Tat of HIV-1 interferes with intracellular function by evading host response mechanisms, and may, therefore, contribute to the high inflammatory reaction in HIV-infected PE [44]. The Tat protein is a trans-activator of viral gene expression and is released extracellularly during HIV acute infection [45]. Since Tat has a similar arginine- and lysine-rich sequence to VEGF, it is recognized as a powerful angiogenic factor [46]. Tat imitates VEGF by attaching to and stimulating Flk-1/KDR [47]. Tat promotes endothelial cell adhesion through the binding of its arginine–glycine–aspartic acid region to the α_v_β_3_ and α_5_β_1_ integrins and VEGFR-2/KDR via its basic domain [46]. Also, a combined Tat/FGF-2 effect is attributed to fibroblast growth factor (FGF-2), which induces the expression of the α_v_β_3_ and α_5_β_1_ integrins, which aids Tat binding [48].

Additionally, HIV-1 via gp120 binds to heparin sulphate proteoglycans (HSPG) on endothelial cells, amplifying viral infectivity and thereby expediting the release of Tat [49]. Tat induces endothelial cells to migrate, adhere, and grow as a capillary-like network in vitro [50]. HIV Tat was also shown to bind F1k-1/KDR, one of the receptors for VEGF, suggesting an additional mechanism for Tat to exert its angiogenic effect [47].

Defective cell signaling by the Tat protein alters endothelial cell morphology, gene expression, and survival by stimulating the MAPK pathway. The movement from the gap 0 to gap 1 (G0 to G1) phase of naïve T cells enables productive HIV infection [51]. The HIV-1 Tat protein facilitates MAPK activity by promoting a change from the G0 to G1 phase of naïve T cells, thereby stimulating HIV infection [51].

## 4. Lymphangiogenesis

Lymphatic vessels were first described in the 17th century and consists of a vascular-like network. They play a pivotal role in maintaining tissue fluid homeostasis, transport of proteins, macromolecules, and cells such as leucocytes and activated antigen-presenting cells for immune protection [52]. This vascular-like network consists of a monolayer of blind-ended capillaries transferring “lymph” to the collecting lymphatics. The expansion of new lymphatic vessels from pre-existing ones, called lymphangiogenesis, is controlled mainly by growth factors, i.e., VEGFs such as VEGF-C and its ligand VEGFR-3, VEGF-D [53,54,55], and other factors, i.e., hypoxia-inducible factor 1-α (HIF-1α), the Tie/angiopoietin system, neuropilin-2, and integrin-α_9_ [56,57,58,59,60,61]. However, until recently, there was a paucity of data on the lymphatic profile during pregnancy and in PE [62,63,64].

### 4.1. Lymphatic System in the Placenta

The human placenta is an hemochorial organ and is highly vascularized; yet, there are conflicting reports on the presence of lymphatic vessels in the placenta. However, Gu et al. (2006) [65], Wang et al. (2011) [66], and Liu et al. (2015) [67], as well as our recent observations [68], do not confirm the presence of lymphatic vessels in the placenta. The aforementioned groups instead observed a stromal network immunostained with podoplanin. Lymphangiogenesis was observed at the decidua [68,69,70,71,72] and the uterine wall [64,73].

### 4.2. Lymphangiogenesis and Preeclampsia

In PE, a dysfunctional fluid clearance manifests as an excessive accumulation of interstitial fluid causing edema [74]. B cells, macrophages, and reticular stromal cells activate the production of VEGF A, C, and D, thereby affecting signaling pathways for the induction of lymphangiogenesis [74]. Increased lymphangiogenesis (pro VEGF-C) is a compensatory response to the heightened exaggerated inflammatory state of PE [75,76]. Indeed, VEGF induces lymphangiogenesis [65]. Nevertheless, Shange et al. (2017) reported no significant difference between VEGF-C and D from PE mothers and control [74]. This upregulation of VEGF-C in PE was observed in early-onset PE; however, one needs to note that patients were on dual ARV therapy [73].

Furthermore, hypoxia-inducible factor-1 (HIF-1) plays an important role in the pathogenesis of PE, and indirectly enhances the molecular regulation of VEGF [66,67,77]. The upregulated *HIF-1* gene plays a critical role in the pathogenesis of PE [58,78,79,80] and contributes to the lymphangiogenesis in PE.

### 4.3. Lymphangiogenesis and HIV Infection

At the mucosal level, HIV-1 uses endothelial cell co-receptors CXCR4 and CCR5 before disseminating through lymphatic endothelial channels to the lymph nodes and, thereafter, moving into the general blood circulation. HIV infection plays a crucial role in lymphatic development; nevertheless, its functional integrity is complex and not fully understood. Three HIV-1 proteins, notably the envelope glycoprotein (gp120), transactivator of transcription (Tat), and the matrix protein (p17), may contribute to HIV-associated vascular disorders. HIV-1 gp120 induces apoptosis in endothelial cells. Tat triggers angiogenesis by using the matrix protein p17 [81] to stimulate the endothelin-1/endothelin B receptor axis [82], thereby activating the protein kinase Akt and extracellular signal-regulated kinase (ERK) signaling pathways [66,77,82,83].

The secretory protein (Slit2) and its receptor roundabout protein (Robo4) expressed on endothelial cells also serve to modulate endothelial cell permeability and, hence, have a determinant participation in the pathophysiological mechanism of lymphangiogenesis [84]. Although Slit2/Robo4 interactions are not fully elucidated, a previous study reported an inhibition of VEGF-C and a blockage of VEGFR-3 [85]. Additionally, HIV-1 gp120 leads to hyperpermeability of lymphatic cells in vitro via modulation of fibronectin expression and activation of α_5_β_1_ integrins. On the other hand, Slit2 blocks the interaction between α_5_β_1_ and Robo4, thus inhibiting lymphatic hyperpermeability [81].

This results in an imbalance of the Akt and ERK signaling pathways, which leads to dysregulation of lymphangiogenesis in PE, since it was shown that, during the pathophysiology of PE, there is decreased P13K/Akt signaling [86].

### 4.4. Lymphangiogenesis in the Duration of HAART and the Risk of Preeclampsia

By enhancing pro-inflammatory cytokines and chemokines, HIV-1 infection mimics PE, thereby influencing the prevalence of PE among HIV positive women. The HAART intervention improves endothelial function and decreases the inflammatory milieu of PE. However, that is not evident, as the timing and duration of the HAART is not clear in most the studies. Despite long-term use of HAART improving mortality among HIV positive patients, the morbidity (particularly vascular and metabolic in nature) is still a serious concern [87]. Two HIV-1 proteins seem to undermine the beneficial action of HAART in the restoration of endothelial cell (EC) function: HIV-1 Tat and matrix protein p17, which impair the endothelial cells. A recent study on HAART showed that angiogenesis and lymphangiogenesis are downregulated with Nucleoside reverse transcriptase inhibitors (NRTIs) by inducing mitochondrial oxidative stress and subsequently impairing receptor tyrosine kinase (RTK) signaling in EC [88], suggesting that NRTIs might trigger the development of PE.

The prevalence of PE in HIV-infected pregnancies is lower; however, upon HAART administration, the risk of PE development increases [13,89]. The association between lymphangiogenesis in the duration of HAART and the risk of PE development is unclear; hence, more research on lymphangiogenesis at the maternal and fetal interface is vital, particularly in immune transfer and ARV usage.

## 5. Highly Active Anti-Retroviral Therapy

Protease inhibitors (PI) induce the progression of Kaposi sarcoma [90]. PIs are potent anti-angiogenic factors that block FGF action [91]. PIs deter HIV aspartyl protease and, hence, the production of HIV virions, thus promoting immune restoration. Also, glucose transporter (GLUT)-4, inhibits glucose uptake and affects the cellular proteasome by triggering p53 protein intracellular accumulation, resulting in apoptosis. Finally, the functional impairment of activator protein (AP)-1, specificity protein (SP)-1 or nuclear factor kappa b (NF-κB) transcription factors leads to a decline in MMP and VEGF expression, thereby preventing angiogenesis.

Anti-retroviral drugs regimens are associated with the development of metabolic disorders such as insulin resistance, dyslipidemia, impaired glucose tolerance, and abnormal body fat distribution, which predispose HIV-infected individuals to cardiovascular-related diseases [92]. Anti-retroviral therapy was also shown to lead to endothelial dysfunction [93,94] and decreased nitric oxide, ultimately resulting in induced endothelial oxidative stress [95], which is similarly observed during the pathophysiology of PE [96]. It is, therefore, possible that predisposition to PE may result from endothelial dysfunction and reduced nitric oxide synthase induced by HAART exposure.

Although some studies report on the endothelial HAART-induced endothelial dysfunction, conflicting reports exist. A study done by Torriani et al. (2008) showed improved endothelial function after ARV administration [97]. Additionally, Savvidou et al. (2011) found normal placental perfusion among HIV-infected women, with uncomplicated pregnancies, receiving and not receiving HAART [98]. In contrast, a study done by Sebitloane et al. (2017) evaluating the effect of HAART on HDP showed that, among all women with HIV, a greater risk of mortality due to HDP was reported among those who received HAART compared with those who did not [99].

## 6. Immune Maladaptation

### 6.1. Natural Killer Cells in Normal versus Preeclamptic Pregnancies

Natural killer (NK) cells are dysregulated in the presence of preeclampsia and HIV infection. In normal pregnancy, these cells promote placental development by balancing the immune response at the maternal–fetal interface [100]. The function of NK cells is controlled by inhibitory receptors [101] and activating receptors, C-type lectin receptors, and Ig-like receptors (2B4)] [102,103,104].

During normal pregnancy, the interaction between the maternal NK cells and fetal cells is controlled by NK cell inhibitory receptors, which prevents inadequate trophoblast invasion. However, this action is prevented in PE pregnancies since activating receptors are predominant, leading to shallow trophoblast invasion [105]. Similarly, the function of NK cells during HIV infection is downregulated or similar to NK cells in a healthy pregnancy state [106]. A study conducted by Mela and Goodier showed reduced activation peripheral NK cells of HIV-infected individuals [107].

### 6.2. Role of HAART on NK Cells and Risk of Preeclampsia Development

Natural killer cells play a role in controlling HIV [108] and are also reported to play a role in pregnancy complications such as miscarriage, implantation failure, and PE development [109,110,111]. In the duration of HAART, NK cells control HIV by secreting CC chemokines. These chemokines inhibit HIV replication via activation of non-cytolytic mechanisms [112]. Several studies reported on the influence of changes that may occur on NK cells in the duration of HAART exposure, and found conflicting results. A study by Valentin et al. (2002) reported higher frequency of NK cells after HAART initiation [113]. Similar findings were shown by Ballan et al. (2007) and Michaelsson et al. (2008) [114,115]. In contrast, a study done by Fria et al. (2015) examining the HAART effect on T-cell recovery versus NK cells found low NK subset recovery after HAART exposure when compared with T-cell recovery during the early months of therapy [116], suggesting that HIV infection of NK cells is important for viral persistence [113].

NK cells are also implicated in pregnancy complications; it was documented that NK cell activation may lead to inadequate trophoblast invasion and may result in exaggerative immune response, which is commonly associated with PE development [117]. Therefore, the possible mechanism responsible for PE development in HIV-infected women might be due to T-cell activation rather than NK cell subset recovery. More studies are needed to confirm how NK cells are regulated in the duration of HAART in order to understand the pathogenesis of PE in HIV-associated pregnancies.

## 7. Cytokines in Normal Pregnancy, Preeclampsia, HIV Infection, and in the Duration of HAART

### 7.1. T Helper Cell 1 and T Helper Cell 2 (Th1 and Th2)

**Figure 1:**
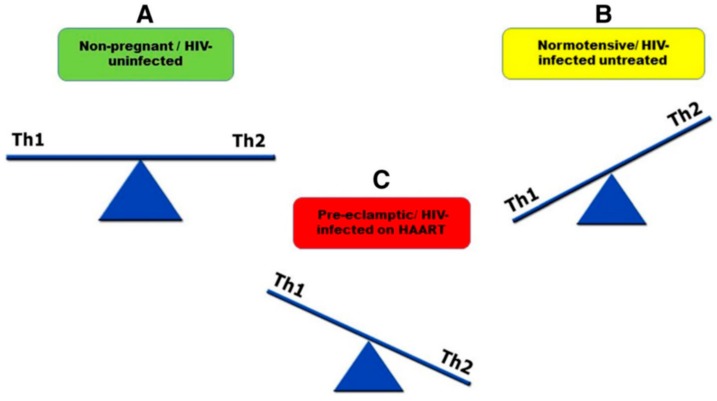
Schematic diagram representing how pro-inflammatory (Th1) and anti-inflammatory (Th2) cytokine are regulated in **A** non-pregnant or HIV-uninfected, **B** normotensive or HIV-infected untreated and **C** pre-eclamptic or HIV-infected on HAART. **A** Shows a balance in the distribution of Th1 and Th2. In **B** there is an imbalance of cytokines with more Th2 release than Th1. This imbalance increases of HIV infection in untreated women. In **C** Th1 levels are higher than Th2. HAART induces Th1 response and leads to pre-eclampsia development [123].

During normal pregnancy, anti-inflammatory (Th2) cytokines are predominant [118], whereas, during the pathogenesis of PE, pro-inflammatory (Th1) cytokines are predominant [119]. However, during the progression of HIV infection, Th2 cytokines are predominant (Figure 1) [120,121]. HIV-infected pregnant women on HAART present a shift toward Th1 immune response [122]. Therefore, HIV-infected pregnant women on HAART have increased risk of developing PE [123].

### 7.2. T Helper Cell 17 (Th17) and T Regulatory Cells (Treg)

Immune cells involved in pregnancy extend from Th1/Th2 into the Th1/Th2/Th17 and regulatory T cells (Treg), introducing Treg as regulators of Th17 lymphocytes and other immune cell types involved in placental development and maintenance [118,124].

Th17 cells are characterized by the secretion of IL-17/IL-17A and are also associated with inducing Th1 cytokine production. An upregulation of Th17 cells is associated with the pathophysiology of autoimmune, chronic inflammatory diseases, allergic disorders, and graft-rejection reactions [125]. Furthermore, it was reported that Th17 cells are upregulated in PE compared to normotensive pregnancies [126,127] and downregulated during the progression of HIV infection [128]. Currently, no studies investigated how IL-17A is regulated in the presence of both PE and HIV infection; more studies are needed in order to have a better understanding of how this cytokine is regulated in the pathophysiology of both conditions, especially in the duration of HAART.

Regulatory T cells are another type of lymphocytes involved in the pathophysiology of PE. In pregnancy, upregulation of these cells is important for maintaining normal pregnancy development [129,130,131]. Downregulation of Treg cells was reported in PE [132].

In the presence of HIV infection, the frequency of Treg cells is increased, implying their role in the progression of the disease [133,134,135]. In the duration of HAART, the frequency of Treg cells was shown to be decreased or similar to that of HIV-uninfected individuals [136,137]. Currently, there are no studies that investigated how Treg cells are regulated in the presence of both PE and HIV infection. Therefore, more studies are needed in order to improve management of PE in the presence of HIV infection, and in order to have a better understanding of the pathophysiology of PE in the presence of HIV infection.

## 8. Conclusions

This paper elaborated on the paradigm shift of HIV’s effect on angiogenesis in normotensive and preeclamptic pregnancy. Whilst an imbalance in the angiogenic and lymphangiogenic transference predominates in PE, we highlight the parodist effect of HIV as it utilizes its accessory proteins to exploit VEGF’s effect. Furthermore, due to the ubiquitous nature of HIV infection in South Africa, this paper also outlines the effect of HAART on the risk of PE development, albeit not on the duration of the therapy. Current literature is controversial on the effect of HAART on T-cell reconstitution, with regard to NK cell subset recovery and the influence of Th1/Th2/Th17 and Treg cell dysregulation during HIV infection in pregnancy. Since cytokine stimulation is disparate in HIV infection, PE, and during ARV usage, it is important that future research outlines the archetypal effect in pregnancy. Finally, this will improve therapeutic interventions in HIV-associated preeclamptic pregnancies, thus reducing maternal and fetal morbidity and mortality.

## Abbreviations

Ang-1	Angiopoietin-1
Ang-2	Angiopoietin-2
Ang-3	Angiopoietin-3
Ang-4	Angiopoietin-4
AP-1	Activator protein 1
ARV	Anti-retroviral therapy
CD94	Cluster of differentiation 94
CTLA-4	Cytotoxic T-lymphocyte antigen 4
CXCR1	Chemokine (C–X–C motif) receptor 1
CXCR2	Chemokine (C–X–C motif) receptor 2
ENOS	Endothelial nitric oxide synthase
FGF-2	Fibroblast growth factor
FIK-1	Vascular endothelial growth factor receptor 2 (VEGFR22, kinase domain receptor)
FOXP3	Forkhead box P3
Gp120	Glycoprotein 120
HIF-1	Hypoxic-inducible factor 1
HAART	Highly active anti-retroviral therapy
HDP	Hypertensive disorders of pregnancy
HELLP	Hemolysis, elevated liver enzymes, and low platelets
HIV	Human immuno-deficiency virus
IL-17	Interleukin-17
KDR	Kinase insert domain receptor
KIR2DS	Killer-cell immunoglobulin-like receptor 2DS
KZN	KwaZulu-Natal
LAIR-1	Leukocyte-associated immunoglobulin-like receptor 1
LIR1	Leukocyte immunoglobulin-like receptor 1
MAPKs	Mitogen-activated protein kinases
MMP	Matrix metalloproteases
NF-κB	Nuclear factor kappa B
NKG2	Natural killer cell G2
NKG2C	Natural killer cell G2A
NKG2D	Natural killer cell G2D
NKp30	Natural killer cell precursor 30
NKp44	Natural killer cell precursor 44
NKp46	Natural killer cell precursor 46
PE	Preeclampsia
PECAM-1	Platelet endothelial cell adhesion molecule 1
PLGF	Placental growth factor
SEng	Soluble endoglin
SFlt1	Soluble fms-like tyrosine kinase 1
Slit2/Robo4	Slit/Roundabout (Robo)
Sp-1	Specificity protein 1
Tat	Transactivating regulatory protein
TGF-ß	Transforming growth factor beta
TIE1	Tyrosine protein kinase receptor 1
TIE2	Tyrosine protein kinase receptor 2
Th1	T helper cell type 1
Th2	T helper cell type 2
Th17	T helper type 17
Treg	Regulatory T cells
UNAIDS	United Nations Program on HIV/AIDS
VE cadherin	Vascular endothelial cadherin
VEGF	Vascular endothelial growth factor
VEGFR-1	Vascular endothelial growth factor receptor 1
VEGFR-2	Vascular endothelial growth factor receptor 2
VEGFR-3	Vascular endothelial growth factor receptor 3
WHO	World Health Organization

## References

[1] World Health Organization (2016). World Health Statistics 2016: Monitoring Health for the Sdgs Sustainable Development Goals.

[2] Pretoria: National Department of Health (2018). Saving Mothers 2014–2016: Seventh Triennial Report on Confidential Enquiries into Maternal Deaths in South Africa: Executive Summary.

[3] Moodley J. (2007). Maternal deaths due to hypertensive disorders in pregnancy: Saving mothers report 2002–2004. Cardiovasc. J. Afr..

[4] Payne B., Hanson C., Sharma S., Magee L., von Dadelszen P., Magee L.A., von Dadelszen P., Stones W., Mathai M. (2016). Epidemiology of the hypertensive disorders of pregnancy. The FIGO textbook of pregnancy hypertension.

[5] Moodley J., Onyangunga O., Maharaj N. (2016). Hypertensive disorders in primigravid black south african women: A one-year descriptive analysis. Hypertens. Pregnancy.

[6] Khan K.S., Wojdyla D., Say L., Gülmezoglu A.M., van Look P.F. (2006). Who analysis of causes of maternal death: A systematic review. Lancet.

[7] Osungbade K.O., Ige O.K. (2011). Public health perspectives of preeclampsia in developing countries: Implication for health system strengthening. J. Pregnancy.

[8] Lakew Y., Reda A.A., Tamene H., Benedict S., Deribe K. (2013). Geographical variation and factors influencing modern contraceptive use among married women in ethiopia: Evidence from a national population based survey. Reprod. Health.

[9] UNAIDS The Joint United Nations Programme on HIV/AIDS. https://www.unAIDS.org/en.

[10] UNAIDS Global HIV & AIDS Statistics—2018 Fact Sheet. https://www.unAIDS.org/en.

[11] Human Sciences Research Council (2008). South African National HIV Prevalence Incidence and Behaviour Survey.

[12] National Department of Health (2017). National Antenatal Sentinel HIV & Syphilis Survey Report.

[13] Kalumba V.M., Moodley J., Naidoo T.D. (2013). Is the prevalence of pre-eclampsia affected by HIV/AIDS? A retrospective case-control study. Cardiovasc. J. Afr..

[14] Kubis N., Levy B.I. (2003). Vasculogenesis and angiogenesis: Molecular and cellular controls. Part 1: Growth factors. Int. Neuroradiol. J. Perith. Neuroradiol. Surg. Proc. Relat. Neurosci..

[15] Reynolds L.P., Killilea S., Redmer D. (1992). Angiogenesis in the female reproductive system. FASEB J..

[16] Risau W. (1997). Mechanisms of angiogenesis. Nature.

[17] Naicker T., Khedun S.M., Moodley J., Pijnenborg R. (2003). Quantitative analysis of trophoblast invasion in preeclampsia. Acta Obstet. Gynecol. Scand..

[18] Paydas S., Ergin M., Seydaoglu G., Erdogan S., Yavuz S. (2009). Pronostic significance of angiogenic/lymphangiogenic, anti-apoptotic, inflammatory and viral factors in 88 cases with diffuse large b cell lymphoma and review of the literature. Leuk. Res..

[19] Chen J.Y., Ribaudo H.J., Souda S., Parekh N., Ogwu A., Lockman S., Powis K., Dryden-Peterson S., Creek T., Jimbo W. (2012). Highly active antiretroviral therapy and adverse birth outcomes among HIV-infected women in botswana. J. Infect. Dis..

[20] Wimalasundera R., Larbalestier N., Smith J., De Ruiter A., Thom S.M., Hughes A., Poulter N., Regan L., Taylor G. (2002). Pre-eclampsia, antiretroviral therapy, and immune reconstitution. Lancet.

[21] Powis K.M., McElrath T.F., Hughes M.D., Ogwu A., Souda S., Datwyler S.A., von Widenfelt E., Moyo S., Nádas M., Makhema J. (2013). High viral load and elevated angiogenic markers associated with increased risk of preeclampsia among women initiating highly active antiretroviral therapy (haart) in pregnancy in the Mma Bana study, Botswana. J. Acquir. Immune Defic. Syndr..

[22] Conroy A.L., McDonald C.R., Gamble J.L., Olwoch P., Natureeba P., Cohan D., Kamya M.R., Havlir D.V., Dorsey G., Kain K.C. (2017). Altered angiogenesis as a common mechanism underlying preterm birth, small for gestational age, and stillbirth in women living with HIV. Am. J. Obstet. Gynecol..

[23] Nnabuike Chibuoke Ngene J.M.a.T.N. (2019). The performance of pre-delivery serum concentrations of angiogenic factors in predicting postpartum antihypertensive drug therapy following abdominal delivery in severe preeclampsia and normotensive pregnancy. PLoS ONE.

[24] Roberts J.M., Rajakumar A. (2009). Preeclampsia and soluble fms-like tyrosine kinase 1. J. Clin. Endocrinol. Metab..

[25] Masuda Y., Shimizu A., Mori T., Ishiwata T., Kitamura H., Ohashi R., Ishizaki M., Asano G., Sugisaki Y., Yamanaka N. (2001). Vascular endothelial growth factor enhances glomerular capillary repair and accelerates resolution of experimentally induced glomerulonephritis. Am. J. Pathol..

[26] Govender N., Moodley J., Gathiram P., Naicker T. (2014). Soluble fms-like tyrosine kinase-1 in HIV infected pre-eclamptic south african black women. Placenta.

[27] Govender N., Naicker T., Rajakumar A., Moodley J. (2013). Soluble fms-like tyrosine kinase-1 and soluble endoglin in HIV-associated preeclampsia. Eur. J. Obstet. Gynecol. Reprod. Biol..

[28] Perucci L.O., Gomes K.B., Freitas L.G., Godoi L.C., Alpoim P.N., Pinheiro M.B., Miranda A.S., Teixeira A.L., Dusse L.M., Sousa L.P. (2014). Soluble endoglin, transforming growth factor-beta 1 and soluble tumor necrosis factor alpha receptors in different clinical manifestations of preeclampsia. PLoS ONE.

[29] Govender N., Moodley J., Naicker T. (2018). The use of soluble fms-like tyrosine kinase 1/placental growth factor ratio in the clinical management of pre-eclampsia. Afr. J. Reprod. Health.

[30] Bates D.O. (2011). An unexpected tail of vegf and plgf in pre-eclampsia. Biochem. Soc. Trans..

[31] Helmo F.R., Lopes A.M.M., Carneiro A., Campos C.G., Silva P.B., dos Reis Monteiro M.L.G., Rocha L.P., dos Reis M.A., Etchebehere R.M., Machado J.R. (2018). Angiogenic and antiangiogenic factors in preeclampsia. Pathol. Res. Pract..

[32] Cerdeira A.S., Agrawal S., Staff A.C., Redman C.W., Vatish M. (2018). Angiogenic factors: Potential to change clinical practice in pre-eclampsia?. BJOG Int. J. Obstet. Gynaecol..

[33] Ngene N.C., Moodley J. (2018). Role of angiogenic factors in the pathogenesis and management of pre-eclampsia. Int. J. Gynaecol. Obstet. Off. Organ Int. Fed. Gynaecol. Obstet..

[34] Shibuya M. (2013). Vascular endothelial growth factor and its receptor system: Physiological functions in angiogenesis and pathological roles in various diseases. J. Biochem..

[35] Coon B.G., Baeyens N., Han J., Budatha M., Ross T.D., Fang J.S., Yun S., Thomas J.-L., Schwartz M.A. (2015). Intramembrane binding of ve-cadherin to vegfr2 and vegfr3 assembles the endothelial mechanosensory complex. J. Cell Biol..

[36] Sahin S., Ozakpinar O.B., Eroglu M., Tetik S. (2014). Platelets in preeclampsia: Function and role in the inflammation. Clin. Exp. Health Sci..

[37] Thakoordeen S., Moodley J., Naicker T. (2017). Serum levels of platelet endothelial cell adhesion molecule-1 (pecam-1) and soluble vascular endothelial growth factor receptor (svegfr)-1 and-2 in HIV associated preeclampsia. Hypertens. Pregnancy.

[38] Findley C.M., Cudmore M.J., Ahmed A., Kontos C.D. (2007). Vegf induces tie2 shedding via a phosphoinositide 3-kinase/akt–dependent pathway to modulate tie2 signaling. Arterioscler. Thrombo. Vasc. Biol..

[39] Mbhele N., Moodley J., Naicker T. (2017). Role of angiopoietin-2, endoglin, and placental growth factor in HIV-associated preeclampsia. Hypertens. Pregnancy.

[40] Fagiani E., Christofori G. (2013). Angiopoietins in angiogenesis. Cancer Lett..

[41] Findley C.M., Mitchell R.G., Duscha B.D., Annex B.H., Kontos C.D. (2008). Plasma levels of soluble tie2 and vascular endothelial growth factor distinguish critical limb ischemia from intermittent claudication in patients with peripheral arterial disease. J. Am. Coll. Cardiol..

[42] Mazibuko M., Moodley J., Naicker T. (2019). Dysregulation of circulating stie2 and sher2 in HIV-infected women with preeclampsia. Hypertens. Pregnancy.

[43] Chen M.K., Hung M.C. (2015). Proteolytic cleavage, trafficking, and functions of nuclear receptor tyrosine kinases. FEBS J..

[44] Abbas W., Herbein G. (2013). T-cell signaling in HIV-1 infection. Open Virol. J..

[45] Romani B., Engelbrecht S., Glashoff R.H. (2010). Functions of tat: The versatile protein of human immunodeficiency virus type 1. J. Gen. Virol..

[46] Zhou F., Xue M., Qin D., Zhu X., Wang C., Zhu J., Hao T., Cheng L., Chen X., Bai Z. (2013). HIV-1 tat promotes kaposi’s sarcoma-associated herpesvirus (kshv) vil-6-induced angiogenesis and tumorigenesis by regulating pi3k/pten/akt/gsk-3β signaling pathway. PLoS ONE.

[47] Albini A., Soldi R., Giunciuclio D., Giraudo E., Benelli R., Primo L., Noonan D., Salio M., Camussi G., Rock W. (1996). The angiogenesis induced by HIV–1 tat protein is mediated by the flk–1/kdr receptor on vascular endothelial cells. Nat Med..

[48] Alghisi G.C., Rüegg C. (2006). Vascular integrins in tumor angiogenesis: Mediators and therapeutic targets. Endothel. Cell Res..

[49] Crublet E., Andrieu J.P., Vives R.R., Lortat-Jacob H. (2008). The HIV-1 envelope glycoprotein gp120 features four heparan sulfate binding domains, including the co-receptor binding site. J. Biol. Chem..

[50] Barillari G., Sgadari C., Palladino C., Gendelman R., Caputo A., Morris C.B., Nair B.C., Markham P., Nel A., Stürzl M. (1999). Inflammatory cytokines synergize with the HIV-1 tat protein to promote angiogenesis and kaposi’s sarcoma via induction of basic fibroblast growth factor and the αvβ3 integrin. J. Immunol..

[51] Li C.J., Ueda Y., Shi B., Borodyansky L., Huang L., Li Y.-Z., Pardee A.B. (1997). Tat protein induces self-perpetuating permissivity for productive HIV-1 infection. Proc. Natl. Acad. Sci. USA.

[52] Detry B., Bruyère F., Erpicum C., Paupert J., Lamaye F., Maillard C., Lenoir B., Foidart J.-M., Thiry M., Noël A. (2011). Digging deeper into lymphatic vessel formation in vitro and in vivo. BMC Cell Biol..

[53] Zheng W., Aspelund A., Alitalo K. (2014). Lymphangiogenic factors, mechanisms, and applications. J. Clin. Investig..

[54] Norrmén C., Tammela T., Petrova T.V., Alitalo K. (2011). Biological basis of therapeutic lymphangiogenesis. Circulation.

[55] Lohela M., Bry M., Tammela T., Alitalo K. (2009). Vegfs and receptors involved in angiogenesis versus lymphangiogenesis. Curr. Opin. Cell Biol..

[56] Kim H., Kataru R.P., Koh G.Y. (2014). Inflammation-associated lymphangiogenesis: A double-edged sword?. J. Clin. Investig..

[57] Alitalo K., Tammela T., Petrova T.V. (2005). Lymphangiogenesis in development and human disease. Nature.

[58] Zampell J.C., Yan A., Avraham T., Daluvoy S., Weitman E.S., Mehrara B.J. (2012). Hif-1α coordinates lymphangiogenesis during wound healing and in response to inflammation. FASEB J..

[59] Mitchell R.N., Kumar V., Abbas A.K., Fausto N. (2010). Robbins and cotran pathologic basis of disease. Saun.

[60] Jiang W.G., Davies G., Martin T.A., Parr C., Watkins G., Mansel R.E., Mason M.D. (2005). The potential lymphangiogenic effects of hepatocyte growth factor/scatter factor in vitro and in vivo. Int. J. Mol. Med..

[61] Lohela M., Saaristo A., Veikkola T., Alitalo K. (2003). Lymphangiogenic growth factors, receptors and therapies. Thromb. Haemost..

[62] Kajiya K., Hirakawa S., Ma B., Drinnenberg I., Detmar M. (2005). Hepatocyte growth factor promotes lymphatic vessel formation and function. EMBO J..

[63] Cao R., Björndahl M.A., Gallego M.I., Chen S., Religa P., Hansen A.J., Cao Y. (2006). Hepatocyte growth factor is a lymphangiogenic factor with an indirect mechanism of action. Blood.

[64] Naghshvar F., Torabizadeh Z., Moslemi Zadeh N., Mirbaha H., Gheshlaghi P. (2013). Investigating the relationship between serum level of s-met (soluble hepatic growth factor receptor) and preeclampsia in the first and second trimesters of pregnancy. ISRN Obstet. Gynecol..

[65] Gu B., Alexander J.S., Gu Y., Zhang Y., Lewis D.F., Wang Y. (2006). Expression of lymphatic vascular endothelial hyaluronan receptor-1 (lyve-1) in the human placenta. Lymphat. Res. Biol..

[66] Wang Y., Sun J., Gu Y., Zhao S., Groome L.J., Alexander J.S. (2011). D2-40/podoplanin expression in the human placenta. Placenta.

[67] Liu H., Li Y., Zhang J., Rao M., Liang H., Liu G. (2015). The defect of both angiogenesis and lymphangiogenesis is involved in preeclampsia. Placenta.

[68] Cele S., Odun-Ayo F., Onyangunga O., Moodley J., Naicker T. (2018). Analysis of hepatocyte growth factor immunostaining in the placenta of HIV-infected normotensive versus preeclamptic pregnant women. Eur. J. Obstet. Gynecol. Reprod. Biol..

[69] Platonova N., Miquel G., Regenfuss B., Taouji S., Cursiefen C., Chevet E., Bikfalvi A. (2013). Evidence for the interaction of fibroblast growth factor-2 with the lymphatic endothelial cell marker lyve-1. Blood.

[70] Brown H., Russell D. (2013). Blood and lymphatic vasculature in the ovary: Development, function and disease. Hum. Reprod. Update.

[71] Jerman L.F., Hey-Cunningham A.J. (2015). The role of the lymphatic system in endometriosis: A comprehensive review of the literature. Biol. Reprod..

[72] Red-Horse K. (2008). Lymphatic vessel dynamics in the uterine wall. Placenta.

[73] Cao R., Ji H., Feng N., Zhang Y., Yang X., Andersson P., Sun Y., Tritsaris K., Hansen A.J., Dissing S. (2012). Collaborative interplay between fgf-2 and vegf-c promotes lymphangiogenesis and metastasis. Proc. Natl. Acad. Sci. USA.

[74] Shange G.P., Moodley J., Naicker T. (2017). Effect of vascular endothelial growth factors a, c, and d in HIV-associated pre-eclampsia. Hypertens. Pregnancy.

[75] Volchek M., Girling J.E., Lash G.E., Cann L., Kumar B., Robson S.C., Bulmer J.N., Rogers P.A. (2010). Lymphatics in the human endometrium disappear during decidualization. Hum. Reprod..

[76] Lely A.T., Salahuddin S., Holwerda K.M., Karumanchi S.A., Rana S. (2013). Circulating lymphangiogenic factors in preeclampsia. Hypertens. Pregnancy.

[77] Onyangunga O.A., Moodley J., Merhar V., Ofusori D.A., Naicker T. (2016). Lymphatic vascular endothelial hyaluronan receptor-1 immunoexpression in placenta of HIV infected pre-eclamptic women. J. Reprod. Immunol..

[78] Spradley F.T., Palei A.C., Anderson C.D., Granger J.P. (2019). Melanocortin-4 receptor deficiency attenuates placental ischemia-induced hypertension in pregnant rats. Hypertension.

[79] Morfoisse F., Renaud E., Hantelys F., Prats A.-C., Garmy-Susini B. (2014). Role of hypoxia and vascular endothelial growth factors in lymphangiogenesis. Mol. Cell. Oncol..

[80] Tal R. (2012). The role of hypoxia and hypoxia-inducible factor-1alpha in preeclampsia pathogenesis. Biol. Reprod..

[81] Zhang X., Yu J., Kuzontkoski P.M., Zhu W., Li D.Y., Groopman J.E. (2012). Slit2/robo4 signaling modulates HIV-1 gp120-induced lymphatic hyperpermeability. PLoS Path..

[82] Caccuri F., Rueckert C., Giagulli C., Schulze K., Basta D., Zicari S., Marsico S., Cervi E., Fiorentini S., Slevin M. (2014). HIV-1 matrix protein p17 promotes lymphangiogenesis and activates the endothelin-1/endothelin b receptor axis. Arterioscl. Thromb. Vasc. Biol..

[83] Basta D., Latinovic O., Lafferty M.K., Sun L., Bryant J., Lu W., Caccuri F., Caruso A., Gallo R., Garzino-Demo A. (2015). Angiogenic, lymphangiogenic and adipogenic effects of HIV-1 matrix protein p17. Path. Dis..

[84] Park K.W., Morrison C.M., Sorensen L.K., Jones C.A., Rao Y., Chien C.-B., Wu J.Y., Urness L.D., Li D.Y. (2003). Robo4 is a vascular-specific receptor that inhibits endothelial migration. Dev. Biol..

[85] Yu J., Zhang X., Kuzontkoski P.M., Jiang S., Zhu W., Li D.Y., Groopman J.E. (2014). Slit2n and robo4 regulate lymphangiogenesis through the vegf-c/vegfr-3 pathway. Cell Commun. Sign..

[86] Khaliq O.P., Murugesan S., Moodley J., Mackraj I. (2018). Differential expression of mirnas are associated with the insulin signaling pathway in preeclampsia and gestational hypertension. Clin. Exp. Hypertens..

[87] Marincowitz C. (2019). The Effects of HIV-1-Proteins and Antiretroviral Therapy on Aortic Endothelial Cells (Aecs)—A Mechanistic In Vitro Approach. Master’s Thesis.

[88] Song L., Ding S., Ge Z., Zhu X., Qiu C., Wang Y., Lai E., Yang W., Sun Y., Chow S.A. (2018). Nucleoside/nucleotide reverse transcriptase inhibitors attenuate angiogenesis and lymphangiogenesis by impairing receptor tyrosine kinases signalling in endothelial cells. Br. J. Pharm..

[89] Sansone M., Sarno L., Saccone G., Berghella V., Maruotti G.M., Migliucci A., Capone A., Martinelli P. (2016). Risk of preeclampsia in human immunodeficiency virus–infected pregnant women. Obstet. Gynecol..

[90] Krischer J., Rutschmann O., Hirschel B., Vollenweider-Roten S., Saurat J.-H., Pechère M. (1998). Regression of kaposi’s sarcoma during therapy with HIV-1 protease inhibitors: A prospective pilot study. J. Am. Acad. Dermatol..

[91] Sgadari C., Barillari G., Toschi E., Carlei D., Bacigalupo I., Baccarini S., Palladino C., Leone P., Bugarini R., Malavasi L. (2002). HIV protease inhibitors are potent anti-angiogenic molecules and promote regression of kaposi sarcoma. Nat. Med..

[92] Filardi P.P., Paolillo S., Marciano C., Iorio A., Losco T., Marsico F., Scala O., Ruggiero D., Ferraro S., Chiariello M. (2008). Cardiovascular effects of antiretroviral drugs: Clinical review. Cardiovasc. Haematol. Disord. Drug Targets..

[93] Fiala M., Murphy T., MacDougall J., Yang W., Luque A., Iruela-Arispe L., Cashman J., Buga G., Byrns R.E., Barbaro G. (2004). Haart drugs induce mitochondrial damage and intercellular gaps and gp 120 causes apoptosis. Cardiovasc. Toxicol..

[94] Zhong D.-s., Lu X.-h., Conklin B.S., Lin P.H., Lumsden A.B., Yao Q., Chen C. (2002). HIV protease inhibitor ritonavir induces cytotoxicity of human endothelial cells. Arterioscler. Thromb. Vasc. Biol..

[95] Chai H., Yang H., Yan S., Li M., Lin P.H., Lumsden A.B., Yao Q., Chen C. (2005). Effects of 5 HIV protease inhibitors on vasomotor function and superoxide anion production in porcine coronary arteries. J. Acquir. Immune Defic. Syndr..

[96] Aouache R., Biquard L., Vaiman D., Miralles F. (2018). Oxidative stress in preeclampsia and placental diseases. Int. J. Mol. Sci..

[97] Torriani F.J., Komarow L., Parker R.A., Cotter B.R., Currier J.S., Dubé M.P., Fichtenbaum C.J., Gerschenson M., Mitchell C.K., Murphy R.L. (2008). Endothelial function in human immunodeficiency virus-infected antiretroviral-naive subjects before and after starting potent antiretroviral therapy: The actg (AIDS clinical trials group) study 5152’s. J. Am. Coll. Cardiol..

[98] Savvidou M., Samuel M., Akolekar R., Poulton M., Nicolaides K. (2011). First trimester maternal uterine artery doppler examination in HIV—positive women. HIV Med..

[99] Sebitloane H.M., Moodley J., Sartorius B. (2017). Associations between HIV, highly active anti-retroviral therapy, and hypertensive disorders of pregnancy among maternal deaths in south africa 2011–2013. Int. J. Gynecol. Obstet..

[100] Dosiou C., Giudice L.C. (2005). Natural killer cells in pregnancy and recurrent pregnancy loss: Endocrine and immunologic perspectives. Endocr. Rev..

[101] King A., Allan D.S., Bowen M., Powis S.J., Joseph S., Verma S., Hiby S.E., McMichael A.J., Loke Y.W., Braud V.M. (2000). HLA-E is expressed on trophoblast and interacts with CD94/NKG2 receptors on decidual nk cells. Eur. J. Immunol..

[102] Wu J., Lanier L.L. (2003). Natural killer cells and cancer. Adv. Cancer Res..

[103] Cerwenka A., Lanier L.L. (2001). Natural killer cells, viruses and cancer. Nat. Rev. Immunol..

[104] Mandal A., Viswanathan C. (2015). Natural killer cells: In health and disease. Hematol. Oncol. Stem Cell Ther..

[105] Wallace A.E., Fraser R., Cartwright J.E. (2012). Extravillous trophoblast and decidual natural killer cells: A remodelling partnership. Hum. Reprod. Update.

[106] Smith C., Jalbert E., de Almeida V., Canniff J., Lenz L.L., Mussi-Pinhata M.M., Cohen R.A., Yu Q., Amaral F.R., Pinto J. (2017). Altered natural killer cell function in HIV-exposed uninfected infants. Front. Immun..

[107] Mela C.M., Goodier M.R. (2007). The contribution of cytomegalovirus to changes in nk cell receptor expression in HIV-1–infected individuals. J. Infect. Dis..

[108] Alter G., Altfeld M. (2009). Nk cells in HIV-1 infection: Evidence for their role in the control of HIV-1 infection. J. Int. Med..

[109] Sharma S. (2014). Natural killer cells and regulatory t cells in early pregnancy loss. Int. J. Dev. Biol..

[110] Tang A.-W., Alfirevic Z., Quenby S. (2011). Natural killer cells and pregnancy outcomes in women with recurrent miscarriage and infertility: A systematic review. Hum. Reprod..

[111] Hashemi V., Dolati S., Hosseini A., Gharibi T., Danaii S., Yousefi M. (2017). Natural killer t cells in preeclampsia: An updated review. Biomed. Pharmacother..

[112] Kottilil S. (2003). Natural killer cells in HIV-1 infection: Role of nk cell-mediated non-cytolytic mechanisms in pathogenesis of HIV-1 infection. Indian J. Exp. Biol..

[113] Valentin A., Rosati M., Patenaude D.J., Hatzakis A., Kostrikis L.G., Lazanas M., Wyvill K.M., Yarchoan R., Pavlakis G.N. (2002). Persistent HIV-1 infection of natural killer cells in patients receiving highly active antiretroviral therapy. Proc. Natl. Acad. Sci. USA.

[114] Michaëlsson J., Long B.R., Loo C.P., Lanier L.L., Spotts G., Hecht F.M., Nixon D.F. (2008). Immune reconstitution of cd56dim nk cells in individuals with primary HIV-1 infection treated with interleukin-2. J. Infect. Dis..

[115] Ballan W.M., Vu B.-A.N., Long B.R., Loo C.P., Michaëlsson J., Barbour J.D., Lanier L.L., Wiznia A.A., Abadi J., Fennelly G.J. (2007). Natural killer cells in perinatally HIV-1-infected children exhibit less degranulation compared to HIV-1-exposed uninfected children and their expression of KIR2DL3, NKG2C, and NKP46 correlates with disease severity. J. Immun..

[116] Frias M., Rivero-Juarez A., Gordon A., Camacho A., Cantisan S., Cuenca-Lopez F., Torre-Cisneros J., Peña J., Rivero A. (2015). Persistence of pathological distribution of nk cells in HIV-infected patients with prolonged use of haart and a sustained immune response. PLoS ONE.

[117] Bachmayer N., Sohlberg E., Sundström Y., Hamad R.R., Berg L., Bremme K., Sverremark-Ekström E. (2009). Women with pre-eclampsia have an altered NKG2A and NKG2C receptor expression on peripheral blood natural killer cells. Am. J. Reprod. Immunol..

[118] Laresgoiti-Servitje E., Gómez-López N., Olson D.M. (2010). An immunological insight into the origins of pre-eclampsia. Hum. Reprod. Update.

[119] Hu W., Wang H., Wang Z., Huang H., Dong M. (2007). Elevated serum levels of interleukin-15 and interleukin-16 in preeclampsia. J. Reprod. Immunol..

[120] Fiore S., Newell M.-L., Trabattoni D., Thorne C., Gray L., Savasi V., Tibaldi C., Ferrazzi E., Clerici M. (2006). Antiretroviral therapy-associated modulation of th1 and th2 immune responses in HIV-infected pregnant women. J. Reprod. Immunol..

[121] Phoswa W.N., Naicker T., Ramsuran V., Moodley J. (2019). Pre-eclampsia: the role of highly active antiretroviral therapy and immune markers. Inflamm. Res..

[122] Maharaj N.R., Phulukdaree A., Nagiah S., Ramkaran P., Tiloke C., Chuturgoon A.A. (2017). Pro-inflammatory cytokine levels in HIV infected and uninfected pregnant women with and without preeclampsia. PLoS ONE.

[123] Machado E.S., Krauss M.R., Megazzini K., Coutinho C.M., Kreitchmann R., Melo V.H., Pilotto J.H., Ceriotto M., Hofer C.B., Siberry G.K. (2014). Hypertension, preeclampsia and eclampsia among HIV-infected pregnant women from latin america and caribbean countries. J. Infect..

[124] Saito S., Nakashima A., Shima T., Ito M. (2010). Th1/th2/th17 and regulatory *t*-cell paradigm in pregnancy. Am. J. Reprod. Immunol..

[125] Tesmer L.A., Lundy S.K., Sarkar S., Fox D.A. (2008). Th17 cells in human disease. Immunol. Rev..

[126] Darmochwal-Kolarz D., Kludka-Sternik M., Tabarkiewicz J., Kolarz B., Rolinski J., Leszczynska-Gorzelak B., Oleszczuk J. (2012). The predominance of th17 lymphocytes and decreased number and function of treg cells in preeclampsia. J. Reprod. Immunol..

[127] Al-Nafea H.M., Hamdy N.M., Aref N.M. (2017). Evaluation of interleukin 17 level as a prognostic marker in active antiviral treated human immunodeficiency virus in saudi patients. Am. J. Biochem..

[128] Campillo-Gimenez L., Cumont M.-C., Fay M., Kared H., Monceaux V., Diop O., Müller-Trutwin M., Hurtrel B., Lévy Y., Zaunders J. (2010). AIDS progression is associated with the emergence of il-17–producing cells early after simian immunodeficiency virus infection. J. Immunol..

[129] Terness P., Kallikourdis M., Betz A.G., Rabinovich G.A., Saito S., Clark D.A. (2007). Tolerance signaling molecules and pregnancy: Ido, galectins, and the renaissance of regulatory t cells. Am. J. Reprod. Immunol..

[130] Saito S., Shima T., Nakashima A., Shiozaki A., Ito M., Sasaki Y. (2007). What is the role of regulatory t cells in the success of implantation and early pregnancy?. J. Assist. Reprod. Genet..

[131] Saito S., Shiozaki A., Sasaki Y., Nakashima A., Shima T., Ito M., Arck P.C., Elkon K.B., Hasler P., Miyazaki T. (2007). Seminars in immunopathology. Regulatory t Cells and Regulatory Natural Killer (nk) Cells Play Important Roles in Feto-Maternal Tolerance.

[132] Sasaki Y., Darmochwal-Kolarz D., Suzuki D., Sakai M., Ito M., Shima T., Shiozaki A., Rolinski J., Saito S. (2007). Proportion of peripheral blood and decidual cd4+ cd25bright regulatory t cells in pre-eclampsia. Clin. Exp. Immunol..

[133] Andersson J., Boasso A., Nilsson J., Zhang R., Shire N.J., Lindback S., Shearer G.M., Chougnet C.A. (2005). Cutting edge: The prevalence of regulatory t cells in lymphoid tissue is correlated with viral load in HIV-infected patients. J. Immunol..

[134] Suchard M.S., Mayne E., Green V.A., Shalekoff S., Donninger S.L., Stevens W.S., Gray C.M., Tiemessen C.T. (2010). Foxp3 expression is upregulated in cd4+ t cells in progressive HIV-1 infection and is a marker of disease severity. PLoS ONE.

[135] Eggena M.P., Barugahare B., Jones N., Okello M., Mutalya S., Kityo C., Mugyenyi P., Cao H. (2005). Depletion of regulatory t cells in HIV infection is associated with immune activation. J. Immunol..

[136] Pozo-Balado M.M., Martínez-Bonet M., Rosado I., Ruiz-Mateos E., Méndez-Lagares G., Rodríguez-Méndez M.M., Vidal F., Muñoz-Fernández M.A., Pacheco Y.M., Leal M. (2014). Maraviroc reduces the regulatory t-cell frequency in antiretroviral-naive HIV-infected subjects. J. Infect. Dis..

[137] Montes M., Sanchez C., Lewis D.E., Graviss E.A., Seas C., Gotuzzo E., White A.C. (2010). Normalization of foxp3+ regulatory t cells in response to effective antiretroviral therapy. J. Inf. Dis..

